# Crosstalk Between Innate and T Cell Adaptive Immunity With(in) the Muscle

**DOI:** 10.3389/fphys.2020.573347

**Published:** 2020-09-18

**Authors:** Adriana C. Bonomo, Fernanda Pinto-Mariz, Ingo Riederer, Claudia F. Benjamim, Gillian Butler-Browne, Vincent Mouly, Wilson Savino

**Affiliations:** ^1^Laboratory on Thymus Research, Oswaldo Cruz Institute, Oswaldo Cruz Foundation, Rio de Janeiro, Brazil; ^2^National Institute of Science and Technology on Neuroimmunomodulation (INCT-NIM), Oswaldo Cruz Institute, Oswaldo Cruz Foundation, Rio de Janeiro, Brazil; ^3^Rio de Janeiro Research Network on Neuroinflammation (RENEURIN), Oswaldo Cruz Institute, Oswaldo Cruz Foundation, Rio de Janeiro, Brazil; ^4^Marzagão Gesteira Institute of Pediatrics, Federal University of Rio de Janeiro, Rio de Janeiro, Brazil; ^5^School of Pharmacy and Biomedical Sciences, University of Central Lancashire, Preston, United Kingdom; ^6^Program of Immunobiology, Institute of Biophysics Carlos Chagas Filho, Federal University of Rio de Janeiro, Rio de Janeiro, Brazil; ^7^Sorbonne Université, Inserm, Institut de Myologie, U974, Center for Research in Myology, Paris, France

**Keywords:** myoblasts, macrophages, dendritic cells, effector T cells, regulatory T cells, polymyositis, Duchenne muscular dystrophy

## Abstract

Growing evidence demonstrates a continuous interaction between the immune system and the skeletal muscle in inflammatory diseases of different pathogenetic origins, in dystrophic conditions such as Duchenne Muscular Dystrophy as well as during normal muscle regeneration. Although one component of the innate immunity, the macrophage, has been extensively studied both in disease conditions and during cell or gene therapy strategies aiming at restoring muscular functions, much less is known about dendritic cells and their primary immunological targets, the T lymphocytes. This review will focus on the dendritic cells and T lymphocytes (including effector and regulatory T-cells), emphasizing the potential cross talk between these cell types and their influence on the structure and function of skeletal muscle.

## Introduction

The Interactions between the immune system and the skeletal muscle in different pathological conditions such as inflammatory myopathies and muscular dystrophies represent a growing field of investigation. Although the macrophage, one important component of innate immunity has been extensively studied in different aspects of muscle regeneration, as well as adjuvants for cell or gene therapy (Chazaud, 2020), much less in known about DC and its partner, the T cell.

During muscle repair macrophages were shown to acquire first a pro-inflammatory and then an anti-inflammatory/regulatory profile, crucial for the maintenance and resolution of the inflammatory process.

It is also known that during muscle repair, the differentiation of muscle progenitors into mature muscle fibers involves a complex orchestration of different cell-cell and cell-matrix interactions as well as interaction with many soluble molecules secreted by these different components present within the regenerating muscle environment. Curiously, many of these mediators are molecules shared with the immune system (Tacke et al., 2009; Costamagna et al., 2015; Sharma and Rudra, 2018). In fact, the cytokines produced by cells of the immune system are not immune-exclusive and many of them are essential for the homeostasis of different tissues. Healing processes in general, use immune mediators as messengers able to reorganize the tissue back into a homeostatic steady state (Tacke et al., 2009; Costamagna et al., 2015; Sharma and Rudra, 2018). However, the end point of the healing process is not always favorable, and fibrosis may occur, precluding healthy tissue from re-emerging. The differences in the possible outcome, healthy vs. fibrotic tissue is strongly dependent upon the correct spatial and temporal secretion of these cytokines.

In healthy muscle, innate immune cells, mostly macrophages, sit between the fibers, in the epimysium and perimysium. These macrophages can be viewed as scavengers for debris, but also as helpers, contributing to muscle formation/regeneration and homeostasis (Lichtman et al., 2016; Zhang et al., 2017; Smigiel and Parks, 2018). When any kind of injury hits a tissue, including muscle, neutrophils are the first cells to migrate in, attracted by chemokines, particularly IL-8, produced by the lesioned endothelium (Perobelli et al., 2015; Silva-Barbosa et al., 2015; Ley et al., 2018).

Very early after injury, resident macrophages and incoming neutrophils are activated by a number of inflammatory stimuli including Danger Associated Molecular Patterns (DAMPs) from pathogens or mammalian cells themselves. DAMPs can trigger a competent immune response (Matzinger, 1994; Matzinger, 2002) after binding to the cognate receptors, Pathogen Recognition Receptors (PRR) present on innate immune cells and on various other cell types (Medzhitov et al., 1997; Medzhitov and Janeway, 2002). The prototypical PRRs are the very conserved family of Toll Like Receptors (TLR),a transmembrane protein first known to regulate caudo-ventral orientation of drosophila embryogenesis. Interestingly, when the TOLL molecule is disrupted, it causes a morphological defect, but more importantly, the flies die of fungal infection indicating that TOLL is also involved in immunity (Lemaitre et al., 1996). The major cell types expressing TLRs are the antigen presenting cells: dendritic cells, macrophages and activated B cells. Yet, other cells of the immune system and others, including muscle, also carry them (Trinchieri and Sher, 2007; Tournadre et al., 2010). More than ten different types of TLRs have been described in mammals, recognizing molecules characteristic of different pathogens or of internal damage. The intracellular or cytoplasmic domain is conserved between TLRs and interleukin-1 family of receptors (IL-1R) and as consequence of activation, these receptors elicit a very potent inflammatory reaction in general (Lemaitre et al., 1996; Medzhitov et al., 1997; Medzhitov and Janeway, 2002).

In the case of an injured muscle, DAMPs derived from the necrotic fiber and endothelial cells, and include Histidyl- tRNA synthetase (HRS), High Mobility Group1 Binding protein (HMGB1) and extracellular ATP. The HMGB1 protein, for example, binds to its specific PRR, TLR4 (Sciorati et al., 2016). Macrophages and dendritic cells, which both express TLR4, are activated in such a way that they, not only produce a number of proinflammatory cytokines (TNF-α, IL-1, IFN-α, IFN-β), but also activate oxidative stress and nitric oxide production, becoming competent antigen presenting cells (Mogensen, 2009; Kawai and Akira, 2010; Cheng et al., 2015; Liu and Cao, 2016). This means that if they meet T lymphocytes, which can recognize the antigen on their surface complexed to MHC molecules, the T cells will become activated. As we will describe in this review, T lymphocytes together with macrophages and DCs, are all found in inflammatory muscle tissue, inflammatory myopathies such as myositis and genetically inherited degenerative diseases of the skeletal muscle, such as Duchenne muscular dystrophy (DMD) (Deyhle and Hyldahl, 2018; Sass et al., 2018). We will provide evidence that connects macrophages, DCs, T effector and T regulatory cells and their secreted molecules. The discussion includes, not only a description of those interactions, which are mediated by the major histocompatibility complex (MHC) and the T-cell receptor, but also cell-cell and cell-matrix interactions, in a molecular context of self-within-self recognition (de Sousa et al., 1991).

### Macrophages: Role in Muscle Regeneration

Macrophages are professional phagocytic cells, since they have a high capacity to eliminate dead and apoptotic cells, cell debris as well as a large number of pathogens. In addition, they produce Reactive Oxygen Species (ROS), secrete soluble factors such as cytokines and chemokines; and present antigens to T lymphocytes. Moreover, macrophages participate in the maintenance of tissue homeostasis and develop specialized functions in a tissue dependent manner (Medzhitov, 2010; Varol et al., 2015). These cells have a remarkable plasticity to adapt to the milieu they are in. Accordingly, they can acquire a pro-inflammatory or an anti-inflammatory/regulatory profile, crucial for the maintenance and resolution of any inflammatory process. In short, inflammatory components such as LPS and cytokines like IL-12, IFN-γ, and TNF-α polarize monocytes and macrophages into a pro-inflammatory population (also known as M1 or classically activated macrophages). Conversely, immunocomplexes, glucocorticoids, and cytokines such as IL-4, IL-13, and IL-10 can induce subpopulation of anti-inflammatory macrophages (known as M2, alternatively activated or regulatory macrophages) (Italiani and Boraschi, 2014; Murray et al., 2014; Vannella and Wynn, 2017; Jurberg et al., 2018).

As previously described, muscle repair is characterized by inflammation (Tidball, 1995; Saclier et al., 2013). It has been shown that pro-inflammatory macrophages co-localize with proliferating myoblasts whereas macrophages expressing anti-inflammatory markers (appearing concomitantly during muscle regeneration) are preferentially associated with myogenin-positive differentiated myoblasts (Lepper et al., 2011). Although Pax7^+^ satellite cells are the only muscle stem cell responsible for muscle regeneration (Warren et al., 2004; Murphy et al., 2011), efficient muscle regeneration has been shown to depend on signaling from other cell types, in particular macrophages. For example, Ly6C^+^CCR2^+^ monocytes differentiate into different macrophages that directly support myogenesis, protect muscle precursor cells from death, and stimulate myoblast proliferation, differentiation, and fusion. Moreover, strategies that prevent the arrival of monocytes into the skeletal muscle after tissue injury result in impaired tissue repair (Cantini et al., 1994; Warren et al., 2005; Summan et al., 2006; Arnold et al., 2007; Contreras-Shannon et al., 2007; Martinez et al., 2010).

It has also been shown that co-cultures of macrophages with myoblasts or macrophage-derived conditioned medium, stimulate myoblast proliferation and delay their differentiation (Johnson and Allen, 1990; Merly et al., 1999; Arnold et al., 2007; Bencze et al., 2012). Furthermore, several pro-inflammatory mediators, including TNF-α, IL-1β, IL-6, HGF, and IGF-I, have been shown to promote myoblast proliferation (Sheehan and Allen, 1999; Li, 2003; Zhang et al., 2013; González et al., 2017; Chaweewannakorn et al., 2018). In particular, HGF has been shown to enhance the migration of human myoblast *in vitro*. This effect is potentiated by the presence of extracellular matrix (ECM) proteins, with the involvement of matrix metalloproteinases and the MAPK/ERK pathways (Zhang et al., 2013). Interestingly the direct contact between macrophages and myogenic cells protects myoblasts and myotubes from cell death; this cell-cell contact being mediated by cell adhesion interactions, including VCAM-1/VLA-4, ICAM-1/LFA-1, and CX3CL1/CX3CR1 (Sonnet et al., 2006; Lesault et al., 2012).

Overall, these data highlight the importance of macrophages as myoblast supporting cells in the regeneration processes (Chazaud et al., 2003; Briggs and Morgan, 2013).

### Dendritic Cells and Muscle Inflammation

Dendritic cells (DCs) are at the interface between innate and adaptive immunity. They are professional antigen-presenting cells (APCs); being known as sentinels of the immune system. They are resident components in non-lymphoid and lymphoid tissues where they take up antigens, migrate into draining lymph nodes, and trigger antigen-specific T and B cell responses (Padilla and Reed, 2008; Hubert et al., 2019). Upon maturation, DCs up-regulate co-stimulatory molecules and MHC Class II molecules (HLA-DR) and secrete a variety of cytokines. Depending on the tissue, the pathogen and the microenvironment, DCs promote a specific and adequate immune response toward Th1, interferon-γ (IFN-γ) secreting T lymphocytes, or other T cell phenotypes like Th2, Th17, or even Treg lymphocytes. Moreover, DCs play a crucial role in activating CD4^+^ and CD8^+^ T cells, B lymphocytes (toward autoantibody production), as well as providing different patterns of cytokine secretion depending on the environment and stimulus they receive (Hubert et al., 2019).

Using a murine model of muscle regeneration, it was demonstrated that MHCII^+^ DCs and macrophages are present within uninjured muscle, and after a transverse crush injury in both anterior tibialis muscle, those cells increased and remained high until day 6 (Pimorady-Esfahani et al., 1997). This data is in agreement with another study, also in a myoinjury model induced by injection of notexin into the anterior tibialis and paravertebral muscle in mice. The authors showed that after injury, the resident macrophages recruit neutrophils and monocytes from the blood, which are progressively substituted by inflammatory DC’s in the regenerating muscle (Brigitte et al., 2010). Both macrophages and inflammatory DCs are important in the response to muscle injury by recognition of self-molecules (for example HMGB-1, SAA1, HSP, DNA, RNA) released from damage cells through TLRs (TLR2/4 and TLR7/8), thus triggering inflammation and tissue repair. However, in dystrophies and myositis, the overwhelming release of cytokine and over-active TLRs can lead to chronic and destructive inflammation (De Paepe, 2020). As such, DCs can be placed as relevant innate immune cells in the context of skeletal muscle inflammation.

Despite the suggested role in a regeneration model, where macrophages seem to have a more relevant and understood role, DCs seems to have a greater participation in Idiopathic Inflammatory Myositis (IIMS).

Idiopathic inflammatory myositis (IIMs) corresponds to a heterogeneous family of diseases with a chronic or subacute onset, involving immune cells and the injured tissue. More recently, IIMs have been divided into four more clearly defined clinical entities, namely dermatomyositis, inclusion body myositis, immune-mediated necrotizing myopathy, and anti-synthetase syndrome (Mariampillai et al., 2018). Over the past years, many studies have tried to characterize the role of the immune cells in IIMs (Tripoli et al., 2020). The evidence for IIMs being an immune-mediated disease comes from the presence of cellular infiltrates within the muscle biopsies, T cell-mediated myocytotoxicity, autoantibodies in the peripheral blood, and association with MHC class I overexpression (Syed and Tournadre, 2015). IIMs are characterized by high levels of circulating cytokines and chemokines, as well as by inflammatory cellular infiltrates, including macrophages, DCs, CD8^+^ T cells (predominantly affecting the endomysium in polymyositis) and CD4^+^ T cells, which affect the perimysium in dermatomyositis (Wiendl et al., 2005; Huang et al., 2018). Although the involvement of DCs has been reported in IIMs (Greenberg, 2007; Greenberg et al., 2007; Wirsdörfer et al., 2016; Hubert et al., 2019), their exact role has not yet been defined.

Two different types of dendritic cells have been described in the muscle infiltrates of IIM patients, with a predominance of plasmocytoid DCs in dermatomyositis, and monocyte-derived DCs in polymyositis and inclusion body myositis (Pimorady-Esfahani et al., 1997; Wienke et al., 2018).

Interestingly, we have observed that human LPS-activated monocyte derived DCs tightly interact with human myoblasts and myotubes. This interaction seems to trigger myoblast proliferation, migration, and cytokine release and to impair myotube differentiation, thus suggesting that activated DCs inhibit myotube formation and muscle regeneration. A similar effect was observed when myoblasts and myotubes were incubated with TNF-α, IFN-γ, and, TGF-β, suggesting a role of circulating cytokines, in addition to the requirement for cell-cell contact. Moreover, co-injection of human myoblasts and DCs into freeze-injured *tibialis anterior* muscle of immunodeficient mice enhanced human myoblast migration, although the absolute number of human muscle fibers was unchanged (Ladislau et al., 2018), similar to what had been shown for macrophages (Bencze et al., 2012). Similarly, increased numbers of activated DCs are seen in inflamed muscle (Pimorady-Esfahani et al., 1997; Padilla and Reed, 2008; Tournadre and Miossec, 2008) suggesting that DCs may also present antigens to T cells at the site of the lesion during myositis, in addition to the classic antigen-presentation in the draining lymph nodes (Hughes et al., 2016). This could be the trigger for autoantibodies production in some types of IIMs. Interesting, myoblasts and muscle fibers from inflammatory myopathies do express molecules typically expressed by APC and/or T cells, namely ICAM-1, HLA-DR, HLA-ABC, CTLA-4, CD28, BB-1, and B7-H1 increasing the chances of having a positive loop on immune activation within the muscle, with modulation of T cell activation and its fate.

The direct participation of DCs in the pathophysiology of inflammatory myopathies was provided in a murine model of polymyositis in C57BL/6 mice, consisting of the transfer of bone marrow-derived dendritic cells (BMDC) pulsed with a skeletal muscle specific antigen (the HILIYSDV peptide, derived from skeletal muscle C protein fragment). Seven days after immunization, the animals presented muscle lesions, induced by DCs, like the features observed in polymyositis. Importantly, such injury was mediated by CD8^+^ T cells since anti-CD8 (but not by anti-CD4) depleting antibodies suppressed disease progression. (Kohyama and Matsumoto, 1999; Okiyama et al., 2014, 2015).

Studies of DCs in Duchenne muscular dystrophy are much scarcer than those reported for myositis. However, some data point to an important role of DCs, since TLR7 expressed on DCs binds to RNA and triggers cytokine production, enhancing the inflammation/degeneration/regeneration cycle. Among the cytokines released, the transforming growth factor (TGF)-β seems to be strongly induced in symptomatic patients, which would explain the participation of DCs, and their consequent interactions with T cells, keeping a positive feedback loop toward the maintenance of a fibrotic and dysfunctional muscle (Mbongue et al., 2014; Rosenberg et al., 2015).

Lastly, it is worth mentioning that the research about DCs during regeneration, myositis and DMD is complicated due to the small number of these cells in the muscle and that their presence probably occurs at the beginning of the disease development. Since patients generally arrive at the hospital once the disease is already established, possibly the role of DC is not relevant at this late time point.

### T Cells in Idiopathic Inflammatory Myopathies and Duchenne Muscular Dystrophy

As mentioned earlier, immune cellular infiltrates including T cells, DCs and macrophages are present in muscle biopsies of inflammatory muscle diseases (Syed and Tournadre, 2015). In this context, with regard to idiopathic inflammatory myositis, an important participation of CD4^+^ Th1, and Th17 cells, B lymphocytes, CD8^+^ T lymphocytes and type I interferon has been reported (Tournadre and Miossec, 2012; Moran and Mastaglia, 2014; Reed et al., 2015; Crowson et al., 2019; Patwardhan and Spencer, 2019). The mechanisms involved in the pathophysiology of the different IIMs seem to differ. While CD8^+^ T cells seem to be important in the pathogenesis of polymyositis and inclusion body myositis, CD4^+^ T cells and B cells play a predominant role in the pathogenesis of dermatomyositis (Rosenberg et al., 2015; Syed and Tournadre, 2015).

Also, the relevance of cytokines in the skeletal muscle lesions seems to be vary according to the IIMs. While type I interferon has been detected in the muscle fibers of patients with dermatomyositis, as well as in plasmacytoid dendritic cells and in the endothelial cells in capillaries, overexpression of IFN-γ induced genes has been associated with inclusion body myositis (Reed et al., 2015; Crowson et al., 2019; Patwardhan and Spencer, 2019).

In the endomysium of patients with inclusion body myositis, dermatomyositis and polymyositis, the presence of T lymphocytes expressing restricted TCR families, in particular Vα2 and Vβ3, suggests that clones capable of recognizing autoantigens participate in the pathophysiology of these diseases (Lindberg et al., 1994). Similarly, in patients with polymyositis, it was observed that endomysial CD8^+^ T cells surround and invade muscle fibers that express MHC class I antigens, with the consequent release of cytotoxic molecules, tissue destruction and release of autoantigens (Hohlfeld and Engel, 1991; Lindberg et al., 1994; Kohyama and Matsumoto, 1999; Levine et al., 2007; Tournadre and Miossec, 2012; Mbongue et al., 2014; Moran and Mastaglia, 2014; Reed et al., 2015; Rosenberg et al., 2015; Syed and Tournadre, 2015; Patwardhan and Spencer, 2019; Crowson et al., 2019). Moreover, numerous CD4^+^ and CD8^+^ T lymphocytes with the phenotype of terminally differentiated cells have been observed in polymyositis and dermatomyositis patients (Crowson et al., 2019). Such cells revealed a cytotoxic capacity, expression of receptors related to NK cells being potent IFN-γ and TNF producers (Fasth et al., 2009). Moreover, *in vitro* studies have shown that such lymphocyte subpopulations are cytotoxic to myotubes (Loell et al., 2011).

Despite previous studies showing increased frequency of highly differentiated CD8^+^ T cell effector memory and terminally differentiated effector cells in patients with dermatomyositis and polymyositis, a recent study evaluating patients with various inflammatory myopathies, as well as patients with immune-mediated necrotizing myopathy, reported the presence of such a subpopulation only in patients with inclusion body myositis (IBM) (Greenberg et al., 2019), which could justify the resistance of these patients to treatment with corticosteroids, since terminally differentiated effector cells seems to be resistant to corticotherapy (Benveniste and Allenbach, 2019).

In Inclusion Body myositis, it has been proposed that CD8^+^ terminally differentiated memory effector (TEMRA) T cells are involved in the pathophysiology of the disease through mechanisms involving cytotoxic enzymes (perforin and granzyme) as well as being mediated by IFN-γ, leading to an increase in the expression of HLA class I molecules, endoplasmic reticulum stress and proteasome dysfunction, with a consequent induction of rimmed vacuole formation and degenerative features (Arahata and Engel, 1984).

More recently, some studies focusing on polymyositis and dermatomyositis (not including inclusion body myositis) have suggested the participation of Th17 cells and the cytokines IL-17, IL-22, and IL-6 in the pathophysiology of IIMs. Moreover, IL-17A has been reported in the muscle tissue of patients with IIMs and *in vitro* studies suggest that IL-17A could play a role in the pathophysiology through mechanisms involving chemokine upregulation, increased inflammation and decreased cell migration and myogenic differentiation. Although such findings may point to new therapeutic perspectives, some results remain controversial and further work is still needed (Tournadre and Miossec, 2012; Moran and Mastaglia, 2014; Syed and Tournadre, 2015).

Pioneer studies in DMD patients revealed that the intramuscular inflammatory infiltrate is mainly composed by T lymphocytes (especially CD8^+^ T cells) and macrophages (Arahata and Engel, 1984). Since then, several studies have been carried out to clarify the participation of the immune system in the pathophysiology of this disease. In this respect, it has been proposed that the absence of dystrophin and subsequent muscle cell damage, would result in the release of intramuscular antigens that could be specifically recognized by cells of the immune system (Spencer and Tidball, 2001). In such a context, it has also been observed that T lymphocytes present in the muscle tissue of patients with DMD predominantly express TCR Vβ2, and that this is not a characteristic shared by diseases involving inflammation of muscle tissue, since it was not detected in patients with polymyositis (Arahata and Engel, 1984). In addition, most patients with DMD have a conserved sequence of four amino acids in the CDR3 region of the TCR Vβ2, suggesting that the T cells present in the inflammatory infiltrate may recognize a specific muscle antigen (Mantegazza et al., 1993; Gussoni et al., 1994). Moreover, it has also been demonstrated that all DMD muscle fibers that were invaded by CD8^+^ T cells expressed MHC class I molecules on their surfaces (Emslie-Smith et al., 1989). Once activated, cytotoxic CD8^+^ T lymphocytes could then migrate and recognize specific peptides on the surface of muscle fibers triggering the release of perforin, granzyme and TNF-α, resulting in tissue damage.

In a cohort of 75 DMD patients, we have observed that increased percentages of circulating CD4^+^CD49d^+^ and CD8^+^CD49d^+^ T lymphocytes were correlated with a more rapid progression of the disease. Functionally, T cells from the more severely affected patients exhibited higher trans-endothelial and fibronectin-driven migratory responses when compared to healthy individuals (Pinto-Mariz et al., 2015).

**FIGURE 1:**
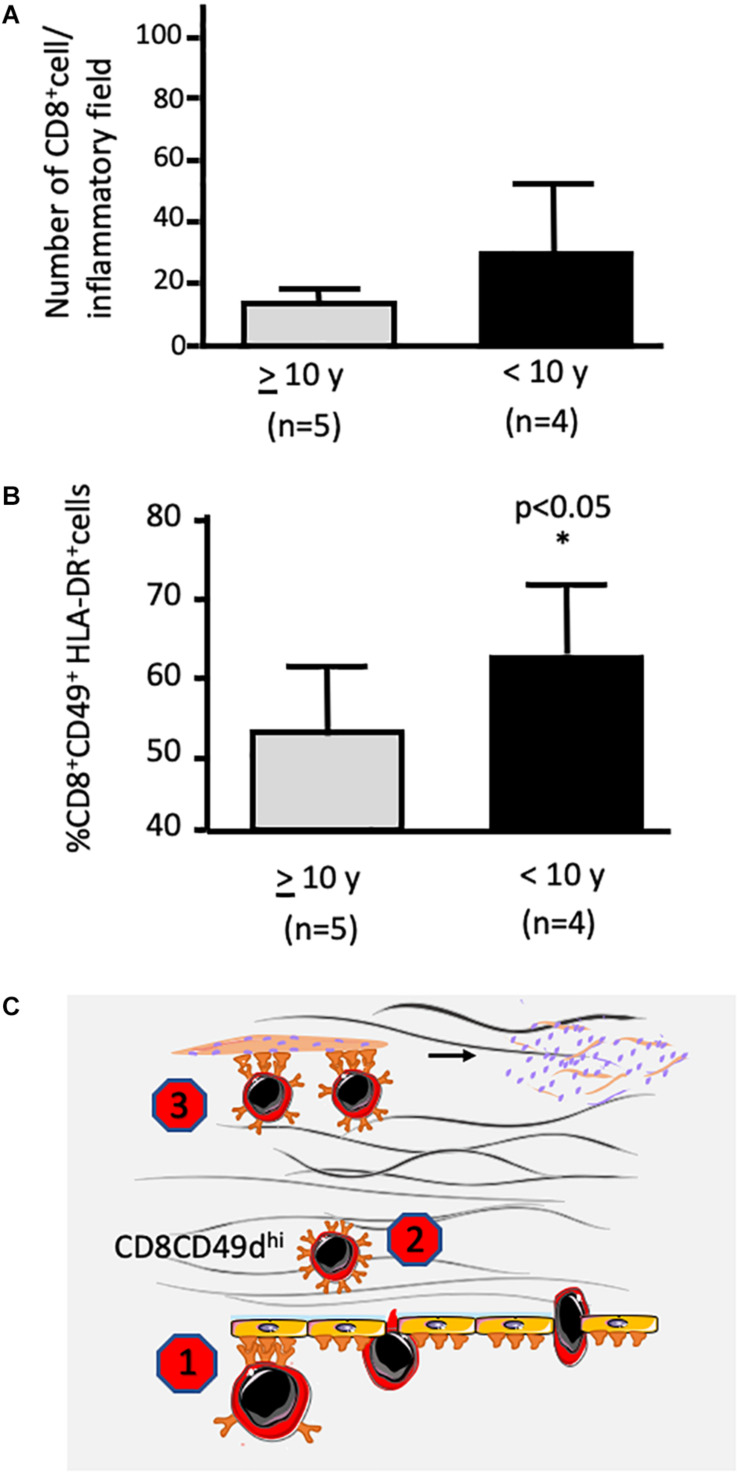
Presence of infiltrating CD8+ T lymphocytes within the muscle of a patient being Duchenne muscular dystrophy: possible inhibition by VLA-4 inhibitors. **(A,B)** reveal CD49d^+^HLA-DR^+^CD8^+^ T cells within the muscular tissue. The biopsies were performed at the onset of the disease. Patients were split according to loss of ambulation before or after 10 years of age. Data were calculated by counting labeled cells by *in situ* immunofluorescence made on frozen sections of muscle biopsies for simultaneous detection of CD8, HLA-DR, and CD49d. **(A)** depicts the numbers of CD8^+^ cells per inflammatory field, whereas **(B)** reveals that the relative numbers of CD49d^+^HLA-DR^+^CD8^+^ T were significantly higher in the muscles from children who lost ambulation before the age of 10 years (slightly modified from Pinto-Mariz et al., 2015). **(C)** illustrates the notion that CD49d inhibitors can block the VLA-4 activity on the transendothelial migration of T lymphocytes (Chazaud, 2020), as well as upon the intramuscular migration of the cells through a fibronectin-containing extracellular network, depicted as pink molecules in the scheme (Sharma and Rudra, 2018). Finally, adhesion of CD49d^high^ T cells to myoblasts as well as myotubes can also be abrogated by an anti-CD49d monoclonal antibody (Tacke et al., 2009), by consequence inhibiting muscle cell apoptosis. * indicates significant difference with *p* < 0.05.

We also observed a higher expression of fibronectin in the muscle of DMD patients, especially those with a worse prognosis, that is, those who lost the ability to walk before the age of ten. More importantly, both CD49^+^CD4^+^CD3^+^ and CD49^+^CD8^+^CD3^+^ were detected within the fibronectin-containing network of the injured muscle (Figure 1). Considering the haptotactic role of fibronectin on T lymphocytes (further enhanced upon activation), the increased production of this protein in muscle tissue of dystrophic patients could enhance the recruitment of more activated T cells toward the lesion. In fact, CD49d^+^ T cell subsets were found in muscular inflammatory infiltrates, and a higher number of activated CD49d^+^HLA-DR^+^CD8^+^ T lymphocytes in the muscle of patients who had a rapid disease progression (Figure 1). Moreover, a higher adhesion of cells obtained from DMD patients to myotubes were observed when compared to healthy controls (Pinto-Mariz et al., 2015).

The importance of CD49d^high^ T lymphocytes in the pathogenesis of the disease has also been observed in the most relevant animal model for this human disease, namely the Golden Retriever muscular dystrophy dog (GRMD). An elevation in the number of circulating CD4^+^CD49d^high^ T cells at early stages of the disease seems to be highly predictive for the loss of ambulation before 6 months of age (Barthélémy et al., 2014). The same result was not observed with CD8^+^CD49d^high^ T cells, suggesting that some mechanisms in GRMD may be different from those involved in DMD human patients. In any case, this is an important finding for stratifying these animals for pre-clinical therapeutic studies.

With regard specifically to muscle tissue, an increase in the gene expression of interferon-γ, TGF-β and chemokines, such as CCL14, CCL2, CXCL-12, and CXCL-14 in patients with DMD has been shown (Evans et al., 2009). Considering that extracellular matrix (ECM) elements can interact directly with immune cells also functioning as a substrate for binding soluble factors such as cytokines and chemokines, we could suggest that the combined action between the increase in fibronectin expression, associated with the rise in some chemokines and cytokines could lead to increased recruitment of inflammatory cells to this area. This would result in a perpetual cycle of inflammatory infiltration and deposition of ECM elements, these events being more prominent in the most severe patients.

Considering the studies performed in DMD patients, it is conceivable that the muscle damage initially caused by the absence of dystrophin could result in exposure of antigens on the surface of muscle fibers. Activated T lymphocytes with high expression of CD49d on the surface could migrate to muscle tissue directed by a chemotactic/haptotactic gradient. Moreover, we demonstrated that CD49d^hi^ T cell subsets obtained from DMD patients have a higher *in vitro* migration capacity across endothelial layers and through fibronectin, when compared to CD49d^low^ T subpopulations (Pinto-Mariz et al., 2015). Once in the tissue, CD8^+^CD49d^hi^ T lymphocytes could recognize antigens on the surface of the fibers causing their destruction.

The higher relative numbers of CD49d^hi^ T cell subsets in the blood of more severe patients, associated with an elevated migratory responsiveness, together with a higher expression of fibronectin and activated CD8^+^ T cells in the muscle, could explain in part the early loss of gait observed in this group of patients.

Overall, effector T cells are important in DMD pathophysiology and evolution with a special role for the CD49d^high^ T cell subpopulations. In this context, CD49d^high^ cells can be used as a prognostic marker of disease progression and CD49d inhibitors can be envisioned as a therapeutic approach to decrease inflammation-mediated tissue damage (see Figure 1), with consequent amelioration in the quality of life in DMD patients. As a hope for treating DMD, a clinical trial using siRNA to inhibit CD49d expression in T cells in DMD patients between 10 and 18 years old with loss of deambulation is ongoing and already in phase II^1^.

### Immune Cross Talk in Skeletal Muscle: The Role of TREG CELLS

As stated above he crosstalk between T cells and macrophages/dendritic cells is essential for immune activation and its maintenance. Although, when shifted to type 1/Th1 immune responses it has a damaging aspect, these same signals are crucial for muscle regeneration, and development. However, if the type 1 response (which includes M1 macrophages and Th1 cells) is not resolved, muscle differentiation does not take place and fibrosis is established (Zhang et al., 2014; Tidball, 2017; Muire et al., 2020).

Fortunately, the immune response has an incredible plasticity and an ongoing response, when healthy, stimulates its own regulation and this is what happens in normal muscle regeneration. Immediately after muscle damage, the type 1 response dominates the scene with the arrival of Th1 lymphocytes and a stimulation of proinflammatory cells. In this phase, proliferation of myoblast progenitors and initial differentiation occurs. This response is crucial for the whole regenerative process and depends on T cells. Genetic deletion of CD8α impairs M1 macrophage infiltration, through the absence of CCL2, leading to a defective muscle regeneration (Zhang et al., 2014). These data indicate the important role of chemokines secreted by CD8^+^ T cells upon monocyte/macrophage attraction to the muscle.

Three to 5 days after the initial injury, there is a shift in the response from a type 1 toward a type 2 (M2 macrophages and Th2 cells)/Treg activity, favoring myoblast fusion and myofiber formation (Riederer et al., 2012).

The signals responsible for this shift are not completely clear, but it is evident that M2 macrophages, secreting TGF-β and IL-10, are crucial for myoblast fusion and maturation (Horsley and Pavlath, 2004). Also, other cells, such as eosinophils, migrate to the lesion and through IL-4/IL-13 secretion modulate fibro-adipogenic precursors (FAP) toward myoblast differentiation supporting fibroblasts (Horsley and Pavlath, 2004; Heredia et al., 2013). IL-4 is also known for its direct role on myoblast fusion through IL-4R present on myoblast and nascent myotubes. Interestingly, this cytokine can be secreted, not only by type 2 immune cells, but also by the myoblasts themselves and the nascent fibers, with a dependence on different NFAT family molecules, similar to the biochemical regulation of T cell differentiation and cytokine secretion (Horsley and Pavlath, 2004).

In parallel to the type 2 response shift, regulatory T cells (Treg) migrate to the lesion and dominate the T cell scenario (Burzyn et al., 2013). Treg cells are CD4^+^ T lymphocytes, characterized by the expression of the transcription factor FoxP3, surface expression of CTLA-4, GITR (the glucocorticoid-induced TNF receptor family related protein) and CD25 – the IL-2 receptor alpha chain).

They can be generated in the thymus during T cell ontogeny or induced in the periphery of the immune system. These cells have an inhibitory action over most immune cells (including other T lymphocyte subpopulations, DCs, macrophages, B cells) and their effector function depends on antigen recognition, enabling secretion of cytokines, such as TGF-β and IL-10. Moreover, inhibitory activity dependent on cell-to-cell contact, has been reported in the absence of TGF-β or IL-10 (Thornton and Shevach, 1998; Shevach, 2006; Ring et al., 2010; Zhang et al., 2017), reinforcing the notion that various mechanisms underly Treg effector function.

In human inclusion body myositis, it has been shown that Tregs are present in small numbers within the muscle, suggesting that the disease is associated with limited Treg numbers, impacting on the suppression by regulatory T cells over an ongoing inflammatory response (Prevel et al., 2013; Allenbach et al., 2014). In contrast, in juvenile dermatomyositis and DMD, Tregs seem to be increased in the diseased muscle although they are less effective in suppressing an immune response (Vercoulen et al., 2014; Villalta et al., 2014). In line with these findings, a study using a *Toxoplasma gondii* experimental model has shown that muscle from animals infected with the parasite are rich in Treg cells, and that Treg elimination allows M2 differentiation, with consequent improvement in muscle homeostasis, thus suggesting that during this chronic infectious stimulation Tregs gain a different functional phenotype (Jin et al., 2017). This can be the result of a chronic inflammatory environment where Tregs, under chronic IFN-g stimulation may acquire a Th1/Treg phenotype, contributing to inflammation and tissue damage. Together these results show that evaluating only the numbers of Treg’s is not sufficient to tell if they are functional or not in chronic muscular diseases. Functional assays are therefore necessary to clarify this issue.

The arrival and maintenance of Tregs in the muscle have been shown to depend on at least two different mechanisms. One of them is dependent on the ATP/P2X axis and was shown in the mdx mouse model of DMD. In this study the authors blocked the ATP/P2 × 7 interaction with periodate-oxidized ATP and observed an increased number of Tregs within the injured muscle. Importantly, in the absence of ATP/P2 × 7 signaling, not only the Tregs did arrive in the lesion but muscle damage was reduced, indicating that ATP blocks Treg arrival and in the absence of Treg, muscle damage is more severe (Gazzerro et al., 2015). Much more is known about the IL-33 mediated Treg arrival and maintenance within the muscle. IL-33 is an alarmin produced by FAPs and skeletal muscle stem cell which binds to the interleukin-1 receptor-like 1 protein (also named ST2) present on CD4^+^ T cells (including Tregs), macrophages and FAPs. In fact, FAP-derived IL-33 is crucial for Treg accumulation within the muscle, and consequently, muscle regeneration (Castiglioni et al., 2015). Muscle T regs, characteristically express ST2 and produce amphiregulin (AREG), a pleiotropic molecule with diverse functions in tissue regeneration and immune suppression (Burzyn et al., 2013; Jin et al., 2018). In the muscle, AREG has been shown to be important for skeletal muscle stem cell expansion in response to IL-33. In addition, muscle Tregs can be modulated by IL-33 producing AREG (Burzyn et al., 2013). Tregs are present in the injured muscle from the very first day, peak between days 3 and 5, and remain in the infiltrate as the majority of T cells until the resolution phase, by day 15). In ageing mice, a deficient production of IL-33 has been shown to be correlated with a diminished migration of Tregs into the muscle whereas treatment with exogenous IL-33 restored the Treg influx and improved muscle regeneration (Kuswanto et al., 2016; Jin et al., 2018).

Regulatory T lymphocytes produce several other cytokines, such as TGF-β and IL-10. Treatment with IL-10 or AREG, both of which are muscle Treg-derived products has been shown to improve muscle repair, as ascertained by an increase in satellite cell and myoblast/myotube markers (Jin et al., 2018). These results strongly suggest that the presence of Tregs contributes to muscle repair.

As mentioned at the beginning of this section, T cells, no matter whether they are CD4 or CD8, T helper or regulatory, need to engage their antigen recognition receptor so that they can signal to other cells. To address this question, Diane Mathis’s laboratory showed an increased frequency of one TCR specificity amongst various mouse strains, suggesting that there is an antigen driven accumulation of Tregs in the muscle (Thornton and Shevach, 1998). The same authors made a transgenic mouse carrying this highly frequent TCR (named mTreg24 tg) and showed that Treg accumulation was in fact antigen driven (Cho et al., 2019). More importantly, this model allowed them to study the kinetics of muscle Treg phenotype acquisition with cell transfer experiments. After transfer of the muscle specific T cells to a normal mouse, muscle Treg phenotype (ST2^+^) was only observed within the muscle, while splenic Tregs, even being specific for muscle antigens, showed the expected splenic Treg signature, with no expression of ST2. Moreover, in the *mdx* model for DMD, mTreg24 provides an accelerated rate of regeneration when compared to polyclonal Treg cells (Cho et al., 2019). It should be noted, however, that such muscle T reg specificity has not yet been studied in humans (Vercoulen et al., 2014; Villalta et al., 2014).

In summary it is very clear that Treg lymphocytes play an important role in the injured/regenerating muscle. Not only do they act as immune suppressors over other immune cells, dampening the inflammatory type 1/Th1 response, but they receive signals from macrophages, FAPs and skeletal muscle stem cells, which in turn, seem to shape their modulatory phenotype inside the muscle. In return, a number of cytokines are secreted by Tregs, favoring muscle stem cell proliferation and progression to myoblast differentiation and fusion in regenerating muscle fibers, if the injury is not chronic. Yet, it should be pointed out that the orchestration of all muscle Treg activities depend on Treg specificity, the only way for T cells to interact long enough with its target, in order to trigger a localized response within the tissue.

## Concluding Remarks

**FIGURE 2:**
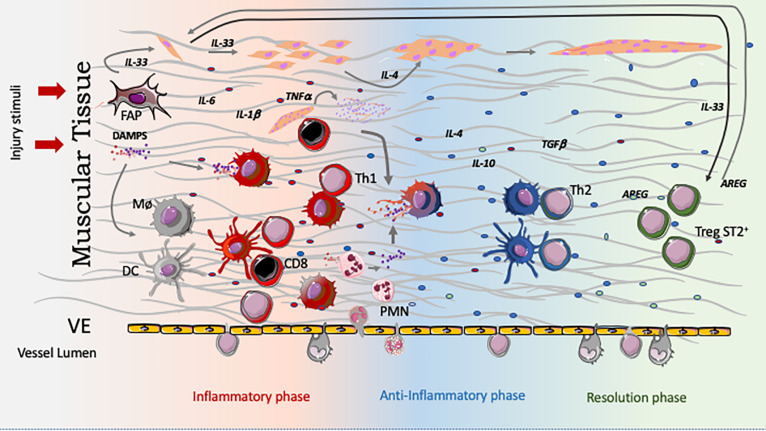
Temporal regulation of the innate and adaptive immune response in muscle injury and healing. Immediately after aggression, muscle cells, as well as fibro-adipogenic precursors (FAPs), produce IL-33. This cytokine induces expansion of the PAX7^+^ satellite cell pool, which themselves also produces IL-33. The necrotic muscle cells release DAMPS, which will stimulate innate immune cells, including resident macrophages and dendritic cells toward a type 1 response represented by the redish colored cells. This initial inflammatory mediator production is guided by cytokines and signals originated from the lesioned muscle. As a result, the whole initial environment is embedded in type 1 cytokines and rapidly incoming cells as neutrophils contribute to the inflammation by several mechanisms. Importantly, after being activated by DAMPs (up to 3 days) both cell types, macrophages and DC, become competent to activate incoming CD4 (Ths) and CD8 T cells keeping, or deviating their function, to a Type 1, building a positive inflammatory loop (this T activation happens in the LN, regardless its possible activation inside the lesioned muscle). Many of the inflammatory cytokines, like TNF-α, IL-1ß, and IL-6 stimulate myoblast progenitor proliferation. After neutrophils come in, attracted by chemokines produced by the vascular endothelium, FAPs and muscle cells themselves, neutrophils are activated, perform their function and evolve to apoptosis. Incoming CD8 cells, besides producing CCL2 which attracts monocytes, will also generate apoptotic bodies by killing damaged muscle cells. Phagocytosis of apoptotic bodies (from neutrophils and muscle) guides macrophages and dendritic cells to a type 2, or type 2 like phenotype(c), contrary to engulfment of necrotic bodies which carry DAMPs. This initial shift is crucial to deviate T cells to a Th2 phenotype. Type 2 responses are linked to IL-4 production which is necessary for myoblast differentiation and fusion. TGF-ß and IL-10 also have roles in the myoblast differentiation and fusion. Interestingly, type one response impairs muscle cell differentiation and maturation. As the healing response progress, together with the shift to type 2 immune response, SP2^+^ Tregs attracted by IL-33, get more numerous and dominates the T cell scenario. Muscle Tregs not only shut down the ongoing immune response but also participate in the healing process, characteristically respond to IL-33 (are ST2^+^ Tregs) producing AREG – a crucial cytokine to expand and maintain the number of satellite cells, in the absence of which regeneration is impaired.

The data discussed above clearly demonstrate the complexity of the cellular and molecular interactions between the skeletal muscle and the immune system, particularly during muscle inflammation. Such a complexity comprises distinct types of interactions, including the production of soluble moieties (cytokines, chemokines), cell-cell interactions mediated by integrin-type ECM receptors, as well as the canonical TCR/MHC-peptide interactions of T lymphocytes with other cells of the immune system or, in certain conditions, with myoblasts themselves. Overall, this complex scenario is schematically depicted in Figure 2.

All these interactions take place independently of a given pathogen-derived antigen, being thus a sort of self-within-self recognition at distinct levels of specificity. Accordingly, we think that it should be necessary to take into account all these interaction levels and specificities, when designing cell or gene therapy strategies for correcting genetic or acquired diseases of the skeletal muscle.

## Author Contributions

AB and WS conceived, participated in writing, and revised the manuscript. CB, FP-M, and IR participated in writing and revised the manuscript. GB-B and VM conceived and revised the manuscript. All authors contributed to the article and approved the submitted version.

## Conflict of Interest

The authors declare that the research was conducted in the absence of any commercial or financial relationships that could be construed as a potential conflict of interest.

## Footnotes

1 http://www.anzctr.org.au/Trial/Registration/TrialReview.aspx?ACTRN=12618000970246

## References

[B1] Allenbach Y. Chaara W. Rosenzwajg M. Six A. Prevel N. Mingozzi F. (2014). Th1 response and systemic treg deficiency in inclusion body myositis. *PLoS One* 9:e88788. 10.1371/journal.pone.0088788 24594700 PMC3942319

[B2] Arahata K. Engel A. G. (1984). Monoclonal antibody analysis of mononuclear cells in myopathies. I: quantitation of subsets according to diagnosis and sites of accumulation and demonstration and counts of muscle fibers invaded by T cells. *Ann. Neurol.* 16 193–208. 10.1002/ana.410160206 6383191

[B3] Arnold L. Henry A. Poron F. Baba-Amer Y. Van Rooijen N. Plonquet A. (2007). Inflammatory monocytes recruited after skeletal muscle injury switch into antiinflammatory macrophages to support myogenesis. *J. Exp. Med.* 204 1057–1069. 10.1084/jem.20070075 17485518 PMC2118577

[B4] Barthélémy I. Pinto-Mariz F. Yada E. Desquilbet L. Savino W. Silva-Barbosa S. D. S. (2014). Predictive markers of clinical outcome in the GRMD dog model of Duchenne muscular dystrophy. *Dis. Model Mech.* 7 1253–1261. 10.1242/dmm.016014 25261568 PMC4213729

[B5] Bencze M. Negroni E. Vallese D. Yacoub-Youssef H. Chaouch S. Wolff A. (2012). Pro-inflammatory macrophages enhance the regenerative capacity of human myoblasts by modifying their kinetics of proliferation and differentiation. *Mol. Ther.* 20 2168–2179. 10.1038/mt.2012.189 23070116 PMC3498804

[B6] Benveniste O. Allenbach Y. (2019). Inclusion body myositis: accumulation of evidence for its autoimmune origin. *Brain* 142 2546–2557. 31497859 10.1093/brain/awz229

[B7] Briggs D. Morgan J. E. (2013). Recent progress in satellite cell/myoblast engraftment – relevance for therapy. *FEBS J.* 280 4281–4293. 10.1111/febs.12273 23560812 PMC3795440

[B8] Brigitte M. Schilte C. Plonquet A. Baba-Amer Y. Henri A. Charlier C. (2010). Muscle resident macrophages control the immune cell reaction in a mouse model of notexin-induced myoinjury. *Arthritis Rheum.* 62 268–279. 10.1002/art.27183 20039420

[B9] Burzyn D. Kuswanto W. Kolodin D. Shadrach J. L. Cerletti M. Jang Y. (2013). A special population of regulatory T cells potentiates muscle repair. *Cell* 155 1282–1295. 10.1016/j.cell.2013.10.054 24315098 PMC3894749

[B10] Cantini M. Massimino M. L. Bruson A. Catani C. Dallalibera L. Carraro U. (1994). Macrophages regulate proliferation and differentiation of satellite cells. *Biochem. Biophys. Res. Commun.* 202 1688–1696. 10.1006/bbrc.1994.2129 8060358

[B11] Castiglioni A. Corna G. Rigamonti E. Basso V. Vezzoli M. Monno A. (2015). FOXP3+ T cells recruited to sites of sterile skeletal muscle injury regulate the fate of satellite cells and guide effective tissue regeneration. *PLoS One* 10:e0128094. 10.1371/journal.pone.0128094 26039259 PMC4454513

[B12] Chaweewannakorn C. Tsuchiya M. Koide M. Hatakeyama H. Tanaka Y. Yoshida S. (2018). Roles of il-1α/β in regeneration of cardiotoxin-injured muscle and satellite cell function. *Am. J. Physiol. Regul. Integr. Comp. Physiol.* 315 R90–R103. 10.1152/ajpregu.00310.2017 29513560

[B13] Chazaud B. (2020). Inflammation and skeletal muscle regeneration: leave it to the macrophages! *Trends Immunol.* 41 481–492. 10.1016/j.it.2020.04.006 32362490

[B14] Chazaud B. Sonnet C. Lafuste P. Bassez G. Rimaniol A. C. Poron F. (2003). Satellite cells attract monocytes and use macrophages as a support to escape apoptosis and enhance muscle growth. *J. Cell Biol.* 163 1133–1143. 10.1083/jcb.200212046 14662751 PMC2173611

[B15] Cheng L. S. Liu Y. Jiang W. (2015). Restoring homeostasis of CD4+ T cells in hepatitis-B-virus-related liver fibrosis. *World J. Gastroenterol.* 21 10721–10731. 10.3748/wjg.v21.i38.10721 26478664 PMC4600574

[B16] Cho J. Kuswanto W. Benoist C. Mathis D. (2019). T cell receptor specificity drives accumulation of a reparative population of regulatory T cells within acutely injured skeletal muscle. *Proc. Natl. Acad. Sci. U.S.A.* 116 26727–26733. 10.1073/pnas.1914848116 31822623 PMC6936428

[B17] Contreras-Shannon V. Ochoa O. Reyes-Reyna S. M. Sun D. Michalek J. E. Kuziel W. A. (2007). Fat accumulation with altered inflammation and regeneration in skeletal muscle of CCR2-/- mice following ischemic injury. *Am. J. Physiol. Cell Physiol.* 292 C953–C967. 10.1152/ajpcell.00154.2006 17020936

[B18] Costamagna D. Costelli P. Sampaolesi M. Penna F. (2015). Role of inflammation in muscle homeostasis and myogenesis. *Mediators Inflamm.* 2015:805172. 10.1155/2015/805172 26508819 PMC4609834

[B19] Crowson C. S. Hein M. S. Pendegraft R. S. Strausbauch M. A. Niewold T. B. Ernste F. C. (2019). Interferon chemokine score and other cytokine measures track with changes in disease activity in patients with juvenile and adult dermatomyositis. *ACR Open Rheumatol.* 1 83–89. 10.1002/acr2.1011 31777784 PMC6857969

[B20] De Paepe B. (2020). Progressive skeletal muscle atrophy in muscular dystrophies-a role for toll-like receptor-signaling in disease pathogenesis. *Int. J. Mol. Sci.* 21:4440. 10.3390/ijms21124440 32580419 PMC7352931

[B21] de Sousa M. Tilney N. L. Kupiec-Weglinski J. W. (1991). Recognition of self within self: specific lymphocyte positioning and the extracellular matrix. *Immunol. Today* 12 262–266. 10.1016/0167-5699(91)90123-b 1910447

[B22] Deyhle M. R. Hyldahl R. D. (2018). The role of T lymphocytes in skeletal muscle repair from traumatic and contraction-induced injury. *Front. Physiol.* 9:768. 10.3389/fphys.2018.00768 29973887 PMC6019499

[B23] Emslie-Smith A. M. Arahata K. Engel A. G. (1989). Major histocompatibility complex class I antigen expression, immunolocalization of interferon subtypes, and T cell-mediated cytotoxicity in myopathies. *Hum. Pathol.* 20 224–231. 10.1016/0046-8177(89)90128-7 2470663

[B24] Evans N. P. Misyak S. A. Robertson J. L. Bassaganya-Riera J. Grange R. W. (2009). Dysregulated intracellular signaling and inflammatory gene expression during initial disease onset in Duchenne muscular dystrophy. *Am. J. Phys. Med. Rehabil.* 88 502–522. 10.1016/j.ajpath.2015.08.010 19454857

[B25] Fasth A. E. Dastmalchi M. Rahbar A. Salomonsson S. Pandya J. M. Lindroos E. (2009). T cell infiltrates in the muscles of patients with dermatomyositis and polymyositis are dominated by CD28null T cells. *J. Immunol.* 183 4792–4799. 10.4049/jimmunol.0803688 19752224

[B26] Gazzerro E. Baldassari S. Assereto S. Fruscione F. Pistorio A. Panicucci C. (2015). Enhancement of muscle T regulatory cells and improvement of muscular dystrophic process in mdx mice by blockade of extracellular ATP/P2X axis. *Am. J. Pathol.* 185 3349–3360. 26465071 10.1016/j.ajpath.2015.08.010

[B27] González M. N. de Mello W. Butler-Browne G. S. Silva-Barbosa S. D. Mouly V. Savino W. (2017). HGF potentiates extracellular matrix-driven migration of human myoblasts: involvement of matrix metalloproteinases and MAPK/ERK pathway. *Skelet Muscle* 7:20. 10.1186/s13395-017-0138-6 PMC5635537 29017538

[B28] Greenberg S. A. (2007). Proposed immunologic models of the inflammatory myopathies and potential therapeutic implications. *Neurology* 69 2008–2019. 10.1212/01.WNL.0000291619.17160.b8 17928577

[B29] Greenberg S. A. Pinkus G. S. Amato A. A. Pinkus J. L. (2007). Myeloid dendritic cells in inclusion-body myositis and polymyositis. *Muscle Nerve* 35 17–23. 10.1002/mus.20649 16969836

[B30] Greenberg S. A. Pinkus J. L. Kong S. W. Baecher-Allan C. Amato A. A. Dorfman D. M. (2019). Highly differentiated cytotoxic T cells in inclusion body myositis. *Brain* 142 2590–2604. 10.1093/brain/awz207 31326977

[B31] Gussoni E. Pavlath G. K. Miller R. G. Panzara M. A. Powell M. Blau H. M. (1994). Specific T cell receptor gene rearrangements at the site of muscle degeneration in Duchenne muscular dystrophy. *J. Immunol.* 153 4798–4805. 7963545

[B32] Heredia J. E. Mukundan L. Chen F. M. Mueller A. A. Deo R. C. Locksley R. M. (2013). Type 2 innate signals stimulate fibro/adipogenic progenitors to facilitate muscle regeneration. *Cell* 153 376–388. 10.1016/j.cell.2013.02.053 23582327 PMC3663598

[B33] Hohlfeld R. Engel A. G. (1991). Coculture with autologous myotubes of cytotoxic T cells isolated from muscle in inflammatory myopathies. *Ann. Neurol.* 29 498–507. 10.1002/ana.410290509 1830466

[B34] Horsley V. Pavlath G. K. (2004). Forming a multinucleated cell: molecules that regulate myoblast fusion. *Cells Tissues Organs* 176 67–78. 10.1159/000075028 14745236

[B35] Huang K. Qiu-Xiang L. Fang-Fang B. I. Hui-Qian D. Mastaglia F. Yue-Bei L. (2018). Comparative immunoprofiling of polymyositis and dermatomyositis muscles. *Int. J. Clin. Exp. Pathol.* 11 3984–3993. 31949787 PMC6962777

[B36] Hubert M. Gobbini E. Bendriss-Vermare N. Caux C. Valladeau-Guilemond J. (2019). Human tumor-infiltrating dendritic cells: from in situ visualization to high-dimensional analyses. *Cancers* 11:E1082. 10.3390/cancers11081082 31366174 PMC6721288

[B37] Hughes C. E. Benson R. A. Bedaj M. Maffia P. (2016). Antigen-presenting cells and antigen presentation in tertiary lymphoid organs. *Front. Immunol.* 7:481. 10.3389/fimmu.2016.00481 27872626 PMC5097899

[B38] Italiani P. Boraschi D. (2014). From monocytes to M1/M2 macrophages: phenotypical vs. functional differentiation. *Front. Immunol.* 5:514. 10.3389/fimmu.2014.00514 25368618 PMC4201108

[B39] Jin R. Blair S. Warunek J. Heffner R. Blader I. J. Wohlfert E. (2017). Regulatory T cells promote myositis and muscle damage in *Toxoplasma gondii* infection. *J. Immunol.* 198 352–362. 10.4049/jimmunol.1600914.109 27895180 PMC5173414

[B40] Jin R. M. Warunek J. Wohlfert E. A. (2018). Therapeutic administration of IL-10 and amphiregulin alleviates chronic skeletal muscle inflammation and damage induced by infection. *Immunohorizons* 2 142–154. 10.4049/immunohorizons.1800024 30417170 PMC6223302

[B41] Johnson S. E. Allen R. E. (1990). The effects of bFGF, IGF-I, and TGF-β on RMo skeletal muscle cell proliferation and differentiation. *Exp. Cell Res.* 187 250–254. 10.1016/0014-4827(90)90088-R 2180733

[B42] Jurberg A. D. Cotta-de-Almeida V. Temerozo J. R. Savino W. Bou-Habib D. C. Riederer I. (2018). Neuroendocrine control of macrophage development and function. *Front. Immunol.* 9:1440. 10.3389/fimmu.2018.01440 29988513 PMC6026652

[B43] Kawai T. Akira S. (2010). The role of pattern-recognition receptors in innate immunity: update on toll-like receptors. *Nat. Immunol.* 11 373–384. 10.1038/ni.1863 20404851

[B44] Kohyama K. Matsumoto Y. (1999). C-protein in the skeletal muscle induces severe autoimmune polymyositis in Lewis rats. *J. Neuroimmunol.* 98 130–135. 10.1016/s0165-5728(99)00087-9 10430046

[B45] Kuswanto W. Burzyn D. Panduro M. Wang K. K. Jang Y. C. Wagers A. J. (2016). Poor repair of skeletal muscle in aging mice reflects a defect in local, interleukin-33-dependent accumulation of regulatory T cells. *Immunity* 44 355–367. 10.1016/j.immuni.2016.01.009 26872699 PMC4764071

[B46] Ladislau L. Portilho D. M. Courau T. Solares-Pérez A. Negroni E. Lainé J. (2018). Activated dendritic cells modulate proliferation and differentiation of human myoblasts. *Cell Death Dis.* 9:551. 10.1038/s41419-018-0426-z 29748534 PMC5945640

[B47] Lemaitre B. Nicolas E. Michaut L. Reichhart J. M. Hoffmann J. A. (1996). The dorsoventral regulatory gene cassette *spatzle/Toll/Cactus* controls the potent antifungal response in Drosophila adults. *Cell* 86 973–983. 10.1016/S0092-8674(00)80172-5 8808632

[B48] Lepper C. Partridge T. A. Fan C. M. (2011). An absolute requirement for pax7-positive satellite cells in acute injury-induced skeletal muscle regeneration. *Development* 138 3639–3646. 10.1242/dev.067595 21828092 PMC3152922

[B49] Lesault P. F. Theret M. Magnan M. Cuvellier S. Niu Y. Gherardi R. K. (2012). Macrophages improve survival, proliferation and migration of engrafted myogenic precursor cells into MDX skeletal muscle. *PLoS One* 7:e46698. 10.1371/journal.pone.0046698 23056408 PMC3462747

[B50] Levine S. M. Raben N. Xie D. Askin F. B. Tuder R. Mullins M. (2007). Novel conformation of histidyl-transfer RNA synthetase in the lung: the target tissue in Jo-1 autoantibody-associated myositis. *Arthritis Rheum.* 56 2729–2739. 10.1002/art.22790 17665459

[B51] Ley K. Hoffman H. M. Kubes P. Cassatella M. A. Zychlinsky A. Hedrick C. C. (2018). Neutrophils: new insights and open questions. *Sci. Immunol.* 3:eaat4579. 10.1126/sciimmunol.aat4579 30530726

[B52] Li Y. P. (2003). TNF-α is a mitogen in skeletal muscle. *Am. J. Physiol. Cell Physiol.* 285 C370–C376. 10.1152/ajpcell.00453.2002 12711593

[B53] Lichtman M. K. Otero-Vinas M. Falanga V. (2016). Transforming growth factor beta (TGF-β) isoforms in wound healing and fibrosis. *Wound Repair Regen.* 24 215–222. 10.1111/wrr.12398 26704519

[B54] Lindberg C. Oldfors A. Tarkowski A. (1994). Restricted use of T cell receptor V genes in endomysial infiltrates of patients with inflammatory myopathies. *Eur. J. Immunol.* 24 2659–2663. 10.1002/eji.1830241114 7957558

[B55] Liu J. Cao X. (2016). Cellular and molecular regulation of innate inflammatory responses. *Cell. Mol. Immunol.* 13 711–721. 10.1038/cmi.2016.58 27818489 PMC5101451

[B56] Loell I. M. Pandya J. Raghavan S. Zong M. Malmstrom V. Lundberg I. E. (2011). Persisting CD28(null) T cells, but not regulatory T cells, in muscle tissue of myositis patients after immunosuppressive therapy. *Arthritis Rheum.* 63 S86–S86.

[B57] Mantegazza R. Andreetta P. Bernasconi F. Baggi F. Oksenberg J. R. Simoncini O. (1993). Analysis of T cell receptor repertoire of muscle-infiltrating T lymphocytes in polymyositis. Restricted V alpha/beta rearrangements may indicate antigen-driven selection. *J. Clin. Invest.* 91 2880–2886. 10.1172/jci116533 8514895 PMC443358

[B58] Mariampillai K. Granger B. Amelin D. Guiguet M. Hachulla E. Maurier F. (2018). Development of a new classification system for idiopathic inflammatory myopathies based on clinical manifestations and myositis-specific autoantibodies. *JAMA Neurol.* 75 1528–1537. 10.1001/jamaneurol.2018.2598 30208379 PMC6583199

[B59] Martinez C. O. McHale M. J. Wells J. T. Ochoa O. Michalek J. E. McManus L. M. (2010). Regulation of skeletal muscle regeneration by CCR2-activating chemokines is directly related to macrophage recruitment. *Am. J. Physiol. Regul. Integr. Comp. Physiol.* 299 R832–R842. 10.1152/ajpregu.00797.2009 20631294 PMC2944434

[B60] Matzinger P. (1994). tolerance, danger, danger, and the extended family. *Annu. Rev. lmmunol.* 12 991–1045. 10.1146/annurev.iy.12.040194.005015 8011301

[B61] Matzinger P. (2002). An innate sense of danger. *Ann. N. Y. Acad. Sci.* 961 341–342. 10.1111/j.1749-6632.2002.tb03118.x 12081934

[B62] Mbongue J. Nicholas D. Firek A. Langridge W. (2014). The role of dendritic cells in tissue-specific autoimmunity. *J. Immunol. Res.* 2014:857143. 10.1155/2014/857143 PMC4022068 24877157

[B63] Medzhitov R. (2010). Inflammation 2010: new adventures of an old flame. *Cell* 140 771–776. 10.1016/j.cell.2010.03.006 20303867

[B64] Medzhitov R. Janeway C. A. (2002). Decoding the patterns of self and nonself by the innate immune system. *Science* 296 298–300. 10.1126/science.1068883 11951031

[B65] Medzhitov R. Preston-Hurlburt P. Janeway C. A. (1997). A human homologue of the *Drosophila* toll protein signals activation of adaptive immunity. *Nature* 388 394–397. 10.1038/41131 9237759

[B66] Merly F. Lescaudron L. Rouaud T. Crossin F. Gardahaut M. F. (1999). Macrophages enhance muscle satellite cell proliferation and delay their differentiation. *Muscle Nerve* 22 724–732. 10.1002/(sici)1097-4598(199906)22:6<724::aid-mus9>3.0.co;2-o 10366226

[B67] Mogensen T. H. (2009). Pathogen recognition and inflammatory signaling in innate immune defenses. *Clin. Microbiol. Rev.* 22 240–273. 10.1128/cmr.00046-08 19366914 PMC2668232

[B68] Moran E. M. Mastaglia F. L. (2014). The role of interleukin-17 in immune-mediated inflammatory myopathies and possible therapeutic implications. *Neuromuscul. Disord.* 24 943–952. 10.1016/j.nmd.2014.06.432 25052503

[B69] Muire P. J. Mangum L. H. Wenke J. C. (2020). Time course of immune response and immunomodulation during normal and delayed healing of musculoskeletal wounds. *Front. Immunol.* 11:1056. 10.3389/fimmu.2020.01056 32582170 PMC7287024

[B70] Murphy M. M. Lawson J. A. Mathew S. J. Hutcheson D. A. Kardon G. (2011). Satellite cells, connective tissue fibroblasts and their interactions are crucial for muscle regeneration. *Development* 138 3625–3637. 10.1242/dev.064162 21828091 PMC3152921

[B71] Murray P. J. Allen J. E. Biswas S. K. Fisher E. A. Gilroy D. W. Goerdt S. (2014). Macrophage activation and polarization: nomenclature and experimental guidelines. *Immunity* 41 14–20. 10.1016/j.immuni.2014.06.008 25035950 PMC4123412

[B72] Okiyama N. Furumoto Y. Villarroel V. A. Linton J. T. Wanxia L. T. Gutermuth J. (2014). Reversal of CD8 T-cell-mediated mucocutaneous graft-versus-host-like disease by the JAK inhibitor tofacitinib. *J. Invest. Dermatol.* 134 992–1000. 10.1038/jid.2013.476 24213371 PMC3961527

[B73] Okiyama N. Hasegawa H. Oida T. Hirata S. Yokozeki H. Fujimoto M. (2015). Experimental myositis inducible with transfer of dendritic cells presenting a skeletal muscle C protein-derived CD8 epitope peptide. *Int. Immunol.* 27 327–332. 10.1093/intimm/dxv00 25577193

[B74] Padilla C. M. L. Reed A. M. (2008). Dendritic cells and the immunopathogenesis of idiopathic inflammatory myopathies. *Curr. Opin. Rheumatol.* 20 669–674. 10.1097/BOR.0b013e3283157538 18946326

[B75] Patwardhan A. Spencer C. H. (2019). Biologics in refractory myositis: experience in juvenile vs. adult myositis; part II: emerging biologic and other therapies on the horizon. *Pediatr. Rheumatol. Online J.* 17:56. 10.1186/s12969-019-0361-2 PMC6702719 31429786

[B76] Perobelli S. M. Galvani R. G. Gonçalves-Silva T. Xavier C. R. Nóbrega A. Bonomo A. (2015). Plasticity of neutrophils reveals modulatory capacity. *Braz. J. Med. Biol. Res.* 48 665–675. 10.1590/1414-431X20154524 26108096 PMC4541684

[B77] Pimorady-Esfahani A. Grounds M. D. McMenamin P. G. (1997). Macrophages and dendritic cells in normal and regenerating murine skeletal muscle. *Muscle Nerve* 20 158–166. 9040653 10.1002/(sici)1097-4598(199702)20:2<158::aid-mus4>3.0.co;2-b

[B78] Pinto-Mariz F. Rodrigues Carvalho L. Prufer De Queiroz Campos A. de Mella W. Ribeiro M. Cabello P. H. (2015). CD49d is a disease progression biomarker and a potential target for immunotherapy in Duchenne muscular dystrophy. *Skelet Muscle* 5:45. 10.1186/s13395-015-0066-2 PMC4674917 26664665

[B79] Prevel N. Allenbach Y. Klatzmann D. Salomon B. Benveniste O. (2013). Beneficial role of rapamycin in experimental autoimmune myositis. *PLoS One* 8:e74450. 10.1371/journal.pone.0074450 24265670 PMC3827074

[B80] Reed A. M. Crowson C. S. Hein M. de Padilla C. L. Olazagasti J. M. Aggarwal R. (2015). Biologic predictors of clinical improvement in rituximab-treated refractory myositis. *BMC Musculoskelet. Disord.* 16:257. 10.1186/s12891-015-0710-3 26382217 PMC4574570

[B81] Riederer I. Negroni E. Bencze M. Wolff A. Aamiri A. Di Santo J. P. (2012). Slowing down differentiation of engrafted human myoblasts into immunodeficient mice correlates with increased proliferation and migration. *Mol. Ther.* 20 146–154. 10.1038/mt.2011.193 21934656 PMC3255588

[B82] Ring S. Karakhanova S. Johnson T. Enk A. H. Mahnke K. (2010). Gap junctions between regulatory T cells and dendritic cells prevent sensitization of CD8(+) T cells. *J. Allergy Clin. Immunol.* 125 237–246.e7. 10.1016/j.jaci.2009.10.025 20109751

[B83] Rosenberg A. S. Puig M. Nagaraju K. Hoffman E. P. Villalta S. A. Rao V. A. (2015). Immune-mediated pathology in Duchenne muscular dystrophy. *Sci. Transl. Med.* 7:299rv4. 10.1126/scitranslmed.aaa7322 26246170 PMC5951380

[B84] Saclier M. Yacoub-Youssef H. Mackey A. L. Arnold L. Ardjoune H. Magnan M. (2013). Differentially activated macrophages orchestrate myogenic precursor cell fate during human skeletal muscle regeneration. *Stem Cells* 31 384–396. 10.1002/stem.1288 23169615

[B85] Sass F. A. Fuchs M. Pumberger M. Geissler S. Duda G. N. Perka C. (2018). Immunology guides skeletal muscle regeneration. *Int. J. Mol. Sci.* 19:835. 10.3390/ijms19030835 29534011 PMC5877696

[B86] Sciorati C. Rigamonti E. Manfredi A. A. Rovere-Querini P. (2016). Cell death, clearance and immunity in the skeletal muscle. *Cell Death Differ.* 23 927–937. 10.1038/cdd.2015.171 26868912 PMC4987728

[B87] Sharma A. Rudra D. (2018). Emerging functions of regulatory T cells in tissue homeostasis. *Front. Immunol.* 9:883. 10.3389/fimmu.2018.00883 29887862 PMC5989423

[B88] Sheehan S. Allen R. (1999). Skeletal muscle satellite cell proliferation in response to members of the fibroblast growth factor family and hepatocyte growth factor. *J. Cell. Physiol.* 181 499–506. 10528236 10.1002/(SICI)1097-4652(199912)181:3<499::AID-JCP14>3.0.CO;2-1

[B89] Shevach E. M. (2006). From vanilla to 28 flavors: multiple varieties of T regulatory cells. *Immunity* 25 195–201. 10.1016/j.immuni.2006.08.003 16920638

[B90] Silva-Barbosa S. D. Butler-Browne G. S. Di Santo J. P. Mouly V. (2015). Comparative analysis of genetically engineered immunodeficient mouse strains as recipients for human myoblast transplantation. *Cell Transplant.* 14 457–467. 10.3727/000000005783982837 16285254

[B91] Smigiel K. S. Parks W. C. (2018). Macrophages, wound healing, and fibrosis: recent insights. *Curr. Rheumatol. Rep.* 20:17. 10.1007/s11926-018-0725-5 29550962

[B92] Sonnet C. Lafuste P. Arnold L. Brigitte M. Poron F. Authier F.-J. (2006). Human macrophages rescue myoblasts and myotubes from apoptosis through a set of adhesion molecular systems. *J. Cell Sci.* 119 2497–2507. 10.1242/jcs.02988 16720640

[B93] Spencer M. J. Tidball J. G. (2001). Do immune cells promote the pathology of dystrophin-deficient myopathies? *Neuromuscul. Disord.* 11 556–564. 11525885 10.1016/s0960-8966(01)00198-5

[B94] Summan M. Warren G. L. Mercer R. R. Chapman R. Hulderman T. Van Rooijen N. (2006). Macrophages and skeletal muscle regeneration: a clodronate-containing liposome depletion study. *Am. J. Physiol. Regul. Integr. Comp. Physiol.* 290 R1488–R1495. 10.1152/ajpregu.00465.2005 16424086

[B95] Syed H. A. Q. Tournadre A. (2015). Idiopathic inflammatory myopathies: from immunopathogenesis to new therapeutic targets. *Rheum. Dis.* 18 818–825. 10.1111/1756-185X.12736 26385431

[B96] Tacke F. Luedde T. Trautwein C. (2009). Inflammatory pathways in liver homeostasis a d liver injury. *Clin. Rev. Allergy Immunol.* 36 4–12. 18600481 10.1007/s12016-008-8091-0

[B97] Thornton A. M. Shevach E. M. (1998). Interleukin 2 production. *J. Exp. Med.* 188 287–296. 9670041 10.1084/jem.188.2.287 PMC2212461

[B98] Tidball J. G. (1995). Inflammatory cell response to acute muscle injury. *Med. Sci. Sports Exerc.* 27 1022–1032. 7564969 10.1249/00005768-199507000-00011

[B99] Tidball J. G. (2017). Regulation of muscle growth and regeneration by the immune system. *Nat. Rev. Immunol.* 17 165–178. 10.1038/nri.2016.150 28163303 PMC5452982

[B100] Tournadre A. Lenief V. Miossec P. (2010). Expression of toll-like receptor 3 and toll-like receptor 7 in muscle is characteristic of inflammatory myopathy and is differentially regulated by Th1 and Th17 cytokines. *Arthritis Rheum.* 62 2144–2151. 10.1002/art.27465 20309865

[B101] Tournadre A. Miossec P. (2008). Chemokine and dendritic cells in inflammatory myopathies. *Ann. Rheum. Dis.* 68 300–304. 10.1136/ard.2008.095984 19213746

[B102] Tournadre A. Miossec P. (2012). Interleukin-17 in inflammatory myopathies. *Curr. Rheumatol. Rep.* 14 252–256. 22350607 10.1007/s11926-012-0242-x

[B103] Trinchieri G. Sher A. (2007). Cooperation of Toll-like receptor signals in innate immune defence. *Nat. Rev. Immunol.* 7 179–190. 10.1038/nri2038 17318230

[B104] Tripoli A. Marasco E. Cometi L. De Stefano L. Marcucci E. Furini F. (2020). One year in review 2019: idiopathic inflammatory myopathies. *Clin. Exp. Rheumatol.* 38 1–10. 32041680

[B105] Vannella K. M. Wynn T. A. (2017). Mechanisms of organ injury and repair by macrophages. *Annu. Rev. Physiol.* 79 593–617. 27959618 10.1146/annurev-physiol-022516-034356

[B106] Varol C. Mildner A. Jung S. (2015). Macrophages: development and tissue specialization. *Ann. Rev. Immunol.* 33 643–675. 25861979 10.1146/annurev-immunol-032414-112220

[B107] Vercoulen Y. Bellutti Enders F. Meerding J. Plantinga M. Elst E. F. Varsani H. (2014). Increased presence of FOXP3+ regulatory T cells in inflamed muscle of patients with active juvenile dermatomyositis compared to peripheral blood. *PLoS One* 9:e105353. 10.1371/journal.pone.0105353 25157414 PMC4144849

[B108] Villalta S. A. Rosenthal W. Martinez L. Kaur A. Sparwasser T. Tidball J. G. (2014). Regulatory T cells suppress muscle inflammation and injury in muscular dystrophy. *Sci. Transl. Med.* 6:258ra142 10.1126/scitranslmed.3009925108 PMC4889432 25320234

[B109] Warren G. L. Hulderman T. Mishra D. Gao X. Millecchia L. O’Farrell L. (2005). Chemokine receptor CCR2 involvement in skeletal muscle regeneration. *FASEB J.* 19 413–415. 10.1096/fj.04-2421fje 15601671

[B110] Warren G. L. O’Farrell L. Summan M. Hulderman T. Mishra D. Luster M. I. (2004). Role of CC chemokines in skeletal muscle functional restoration after injury. *Am. J. Physiol. Cell Physiol.* 286 C1031–C1036. 10.1152/ajpcell.00467.2003 15075201

[B111] Wiendl H. Hohlfeld R. Kieseier B. C. (2005). Immunobiology of muscle: advances in understanding an immunological microenvironment. *Trends Immunol.* 26 374–380. 10.1016/j.it.2005.05.003 15922662

[B112] Wienke J. Deakin C. T. Wedderburn L. R. van Wijk F. van Royen-Kerkhof A. (2018). Systemic and tissue inflammation in juvenile dermatomyositis: from pathogenesis to the quest for monitoring tools. *Front. Immunol.* 9:2951. 10.3389/fimmu.2018.02951 30619311 PMC6305419

[B113] Wirsdörfer F. Bangen J. M. Pastille E. Schmitz D. Flohé S. Schumak B. (2016). Dendritic cell-like cells accumulate in regeneration murine skeletal muscle after injury and boost adaptive immune responses only upon a microbial challenge. *PLoS One* 11:e0155870. 10.1371/journal.pone.0155870 27196728 PMC4873214

[B114] Zhang C. Li L. Feng K. Fan D. Xue W. Lu J. (2017). “Repair” treg cells in tissue injury. *Cell. Physiol. Biochem.* 43 2155–2169. 10.1159/000484295 29069643

[B115] Zhang C. Li Y. Wu Y. Wang L. Wang X. Du J. (2013). Interleukin-6/signal transducer and activator of transcription 3 (STAT3) pathway is essential for macrophage infiltration and myoblast proliferation during muscle regeneration. *J. Biol. Chem.* 288 1489–1499. 10.1074/jbc.M112.419788 23184935 PMC3548462

[B116] Zhang J. Xiao Z. Qu C. Cui W. Wang X. Du J. (2014). CD8 T cells are involved in skeletal muscle regeneration through facilitating MCP-1 secretion and Gr1 high macrophage infiltration. *J. Immunol.* 193 5149–5160. 10.4049/jimmunol.1303486 25339660

